# Unusual Complication of Multidrug Resistant Tuberculosis

**DOI:** 10.1155/2017/6835813

**Published:** 2017-09-18

**Authors:** Prerna Sharma, Ravindra Nath Sahay

**Affiliations:** Division of Medicine, Seth G.S. Medical College & KEM Hospital, Parel, Mumbai 400 012, India

## Abstract

**Introduction:**

Capreomycin is a second-line drug often used for multidrug-resistant tuberculosis which can result in nephrotoxic effects similar to other aminoglycosides. We describe a case of capreomycin induced Bartter-like syndrome with hypocalcemic tetany.

**Case Report:**

23-year-old female patient presented with carpopedal spasms and tingling sensations in hands. Patient was being treated with capreomycin for two months for tuberculosis. On further investigation, hypocalcemia, hyponatremia, hypomagnesemia, hypokalemia, and hypochloremic metabolic alkalosis were noted. Vitamin D and serum PTH levels were within normal limits. Hypercalciuria was confirmed by urine calcium/creatinine ratio. Calcium, potassium, and magnesium supplementation was given and capreomycin was discontinued. Electrolytes normalized in two days after cessation of capreomycin with no further abnormalities on repeat investigations.

**Discussion:**

Aminoglycosides can result in renal tubular dysfunction leading to Fanconi syndrome, Bartter syndrome, and distal tubular acidosis. Impaired mitochondrial function in the tubular cells has been hypothesized as the possible cause of these tubulopathies. Acquired Bartter-like syndrome phenotypically resembles autosomal dominant type 5 Bartter syndrome. Treatment consists of correction of electrolyte abnormalities, indomethacin, and potassium-sparing diuretics. Prompt diagnosis and treatment of severe dyselectrolytemia are warranted in patients on aminoglycoside therapy.

## 1. Introduction

Capreomycin is an aminoglycoside antibiotic indicated in treatment of multidrug-resistant tuberculosis (MDR-TB). With the increase in resistant tuberculosis, capreomycin has been increasingly employed as a second-line drug. Its side-effect profile is essentially similar to other antibiotics in its class. Aminoglycoside-induced nephrotoxicity is attributed to its excellent renal penetration and renal excretion [1]. Although case reports of gentamycin and amikacin induced Bartter syndrome (BS) have been published, there is paucity of data on BS caused by capreomycin. We report a case of hypocalcemic tetany due to BS following capreomycin therapy.

## 2. Case Report

23-year-old female presented to the Emergency Room with carpopedal spasms and tingling numbness in hands. Patient endorsed tingling sensation in hand since a month which was intermittent and unrelated to wrist movement. She denied history of preceding trauma and swelling in hands. There was no history of similar complaints in the past or any prior surgery. She denied complaints like nausea, vomiting, and diarrhea. Tingling in hands was not associated with paresthesia in other extremities and sensory or motor deficits. Patient was a known case of multidrug resistant tuberculosis and was being treated with the following drugs for two months: amoxicillin-clavulanate, ethionamide, intramuscular capreomycin, linezolid, and para-aminosalicylate (PAS) granules. Patient was afebrile and her initial vital signs were normal with a pulse of 78/min and blood pressure of 110/76 mm of Hg. On examination, flattening of chest wall on the left side was noted. Trail's sign was positive with a deviation of the trachea to the left side. On auscultation of lung fields, breath sounds were diminished on the left. These findings suggested presence of tuberculous fibrosis in the left lung. No other stigmata of TB were noted and rest of the physical examination was unremarkable.

Initial blood-work at the time of presentation revealed a low serum calcium level of 6.98 mg/dL. Patient was hospitalized and detailed investigations were done. Coexisting with hypocalcemia, other electrolyte abnormalities noted were as follows: serum sodium of 130 mEq/L, potassium of 1.8 mEq/L, chloride of 95 mEq/L, calcium of 6.98 mg/dL, and magnesium of 0.5 mg/dL. Serum albumin was 4 g/dL. Serum creatinine was normal and remained so throughout the course of hospitalization. The arterial blood gas evaluation showed metabolic alkalosis without respiratory compensation with pH of 7.5, HCO_3_ of 30 mEq/L, and PaCO_2_ of 30 mmHg. Routine urine examination revealed a 2–4 pus cells, 1-2 RBCs, and 1-2 epithelial cells, in the absence of proteinuria and glycosuria. Urine calcium/creatinine ratio was 0.49 (>0.2), which confirmed hypercalciuria. Urinary prostaglandin-E level was not performed as this assay is not readily available in our institute.

Vitamin D and serum parathyroid levels were found to be in the normal range, thus ruling out hypovitaminosis D and secondary hyperparathyroidism, respectively. Patient was treated symptomatically; injectable calcium, magnesium, and potassium were administered to correct electrolyte abnormalities. Due to the association of aminoglycoside antibiotics with alterations in electrolyte levels, capreomycin was discontinued. Subsequently, the electrolytes started to rise after two days of stopping capreomycin (Table 1). Patient was discharged once her symptoms resolved and the serum electrolytes were normalized. On follow-up, there were no complains of tingling sensations or carpopedal spasms and electrolytes remained to be in the normal range.

## 3. Discussion

Bartter syndrome is an inherited renal tubulopathy affecting the loop of Henle (ascending limb) that manifests as hypochloremic metabolic alkalosis, hypokalemia, hypercalciuria, and mild hypomagnesemia. Electrolyte abnormalities similar to that of BS can be caused by chronic diuretic use, vomiting, and therapeutic drugs. Aminoglycosides, amphotericin B, prostaglandins, cisplatin, and heavy metals have been reported to be associated with Bartter-like syndrome [2, 3]. Aminoglycoside antibiotics have also been implicated in the precipitation of renal Fanconi syndrome and distal renal tubular acidosis [4–6].

Nonoliguric renal failure is the most common manifestation of aminoglycoside-induced nephrotoxicity due to its exclusive excretion by the kidneys [1]. Acquired BS is clinically similar to the autosomal dominant phenotypic variant, that is, type 5 Bartter syndrome, which is due to a gain-of-function mutation in the calcium-sensing receptor (CaSR) in the thick ascending limb (TAL) of Henle's loop [6]. Activation of the CaSR receptor by aminoglycosides leads to inhibition of the luminal potassium channels in the TAL [1]. Aminoglycoside-induced impaired mitochondrial protein synthesis and ATP generation have been hypothesized to be the cause of diffuse renal tubular dysfunction [6].

In the case reported above, two-month course of capreomycin caused type 5 Bartter-like electrolyte abnormalities in a female patient on antituberculous therapy. We reported hypochloremic metabolic alkalosis, hypokalemia, hypomagnesemia, hypercalciuria, and hypocalcemia in a normotensive patient which was indicative of Bartter-like syndrome. Capreomycin has been previously reported to be responsible for similar abnormalities [7]; however, hypocalcemic carpopedal spasms due to capreomycin have not been reported, to the best of our knowledge. Hypomagnesemia can also lead to paresthesias and tetany, but BS patients have mild magnesium wasting and unlike patients of Gitelman's syndrome. BS patients have increased urinary prostaglandin-E which would have supplemented our diagnosis had the assay been available; this limited our case report.

Treatment of aminoglycoside-induced BS predominantly consists of symptomatic correction of electrolytes and discontinuation of drug. Indomethacin, potassium-sparing diuretics, and/or aldosterone receptor antagonists have been also recommended [3]. Our patient had a prompt recovery after discontinuing capreomycin and there was no dyselectrolytemia after correction of calcium and potassium. Hypokalemia and hypomagnesemia may result in cardiac arrhythmias and QT-prolongation; drugs prolonging QT-interval must be avoided and prompt treatment of hypokalemia and hypocalcemia is warranted [3]. Patients on antituberculous treatment with capreomycin should be intermittently monitored for acquired renal tubulopathies. Electrolyte abnormalities like hyponatremia and hypokalemia can be potentially life-threatening due to their neurological and cardiac effects, respectively. There is a need for diagnosis and treatment of this essentially reversible condition.

## Figures and Tables

**Table 1:** Evolution of serum electrolytes in acquired Bartter-like syndrome.

Electrolytes (normal range, unit)	Day 0	Day 1	On follow-up
Na^+^ (130–144 mEq/l)	130	132	140
K^+^ (3–4.5 mEq/l)	1.8	2.7	2.6
Ca^++^ (8.4–11 mg/dl)	6.98	6.3	7.8
Mg^++^ (1.6–2.3 mg/dl)	0.5	1.1	1.8
Cl^−^ (96–105 mEq/l)	95	94	102
Phosphorus (2.5–5 mg%)	2.5	2.7	1.8

Na^+^: sodium; K^+^: potassium; Ca^++^: calcium (total); Mg^++^: magnesium; Cl^−^: chloride.
